# Vaginal microbiome community state types and high-risk human papillomaviruses in cervical precancer and cancer in North-central Nigeria

**DOI:** 10.1186/s12885-023-11187-5

**Published:** 2023-07-20

**Authors:** Jonah Musa, Mamoudou Maiga, Stefan J. Green, Francis A. Magaji, Ali J. Maryam, Mark Okolo, Chuwang J. Nyam, Nanma T. Cosmas, Olugbenga A. Silas, Godwin E. Imade, Yinan Zheng, Brian T. Joyce, Brehima Diakite, Imran Morhason-Bello, Chad J. Achenbach, Atiene S. Sagay, Innocent A.O. Ujah, Robert L. Murphy, Lifang Hou, Supriya Dinesh Mehta

**Affiliations:** 1 Department of Obstetrics and Gynecology, College of Health Sciences, University of Jos, Jos, Plateau State Nigeria; 2 Department of Preventive Medicine, Division of Cancer Epidemiology and Prevention, Feinberg School of Medicine, Northwestern University, Chicago, USA; 3 Center for Global Oncology, Institute for Global Health, Feinberg School of Medicine, Northwestern University, Chicago, USA; 4 Center for innovations in Healthcare Technologies, McCormick’s School of Biomedical Engineering, Northwestern University, Chicago, IL USA; 5 Genomics and Microbiome Core Facility, Rush University, Chicago, IL USA; 6 Department of Medical Microbiology, College of Health Sciences, University of Jos, Jos, Nigeria; 7 Division of Epidemiology and Biostatistics, School of Public Health, University of Illinois at Chicago, Chicago, IL USA; 8 Genomics and Postgraduate Core Facility, College of Health Sciences, University of Jos, Jos, Nigeria; 9 Department of Anatomic Pathology and Forensic Medicine, College of Health Sciences, University of Jos, Jos, Nigeria; 10 University of Sciences, Technique and Technologies of Bamako, Bamako, Mali; 11 Department of Obstetrics and Gynecology, College of Medicine, University of Ibadan, Ibadan, Nigeria; 12 Division of Infectious Diseases, Department of Medicine, Feinberg School of Medicine, Northwestern University, Chicago, USA; 13 Robert J. Havey MD, Institute for Global Health, Northwestern University, Chicago, IL USA; 14 Federal University of Health Sciences, Otukpo, Benue State Nigeria; 15 Robert H. Lurie Comprehensive Cancer Center, Department of Preventive Medicine, Northwestern University Feinberg School of Medicine, Chicago, IL 60611 USA; 16 Department of Epidemiology and Biostatistics, Rush University, Chicago, IL USA

**Keywords:** Vaginal microbiome, Cervical precancer and cancer, High-risk HPV, HIV

## Abstract

**Background:**

High risk human papillomaviruses (HR-HPV) have a causal role in cervical oncogenesis, and HIV-mediated immune suppression allows HR-HPV to persist. We studied whether vaginal microbiome community state types (CSTs) are associated with high-grade precancer and/or invasive cervical cancer (HSIL/ICC).

**Methods:**

This was a cross-sectional study of adult women with cervical cancer screening (CCS) at the Jos University Teaching Hospital (JUTH) in Jos, Nigeria, between January 2020 and February 2022. Cervical swabs underwent HPV genotyping (Anyplex™ II HPV28). Cervico-vaginal lavage (CVL) sample was collected for 16 S rRNA gene amplicon sequencing. We used multivariable logistic regression modelling to assess associations between CSTs and other factors associated with HSIL/ICC.

**Results:**

We enrolled 155 eligible participants, 151 with microbiome data for this analysis. Women were median age 52 (IQR:43–58), 47.7% HIV positive, and 58.1% with HSIL/ICC. Of the 138 with HPV data, 40.6% were negative for HPV, 10.1% had low-risk HPV, 26.8% had single HR-HPV, and 22.5% had multiple HR-HPV types. The overall prevalence of any HR-HPV type (single and multiple) was 49.3%, with a higher proportion in women with HSIL/ICC (NILM 31.6%, LSIL 46.5%, HSIL 40.8%, and 81.5% ICC; p = 0.007). Women with HIV were more likely to have HSIL/ICC (70.3% vs. 29.7% among women without HIV). In crude and multivariable analysis CST was not associated with cervical pathology (CST-III aOR = 1.13, CST-IV aOR = 1.31). However, in the presence of HR-HPV CST-III (aOR = 6.7) and CST-IV (aOR = 3.6) showed positive association with HSIL/ICC.

**Conclusion:**

Vaginal microbiome CSTs were not significantly associated with HSIL/ICC. Our findings suggest however, that CST could be helpful in identifying women with HSIL/ICC and particularly those with HR-HPV. Characterization of CSTs using point-of-care molecular testing in women with HR-HPV should be studied as an approach to improve early detection and cervical cancer prevention. Future longitudinal research will improve our understanding of the temporal effect of non-optimal CST, HR-HPV, and other factors in cervical cancer development, prevention, and control.

## Introduction

Approximately 600,000 new cases of invasive cervical cancer (ICC) are diagnosed annually, and over 50% of these women (311,000) die within one year of diagnosis.(1) The high burden of HIV in sub-Saharan Africa and evidence that women living with HIV have 6 times greater risk of developing ICC compared to women without HIV, calls for an urgent focus on prevention and control of cervical cancer in these high-risk populations [1]. Furthermore, epidemiologic studies have confirmed that ICC is primarily caused by human papillomaviruses (HR-HPV) [2] whose persistence and oncogenic potentials are enhanced by HIV-mediated decreases in cellular immunity with other co-factors playing roles in cervical carcinogenesis [3–13].

To improve cervical cancer prevention and control, the WHO launched the cervical cancer elimination initiative in 2018, recommending HPV vaccination, early detection of precancer through screening with a high-performing HPV test, and treatment of precancer and early invasive cancer [14]. The HIV and HR-HPV are sexually transmissible viruses and their attribution to cervical carcinogenesis has led to experts ascribing cervical cancer as a sexually transmissible cancer, [15] and previous study reports have shown that cervico-vaginal microbiome play a significant role in the process [10, 16–18]. Additionally, natural history studies of cervical carcinogenesis have expanded our understanding of the potential mechanism by which vaginal microbiome alone or in conjunction with local pro inflammatory cytokines act either directly or mediated through HR-HPV persistence in advancing progression of cellular changes in a normal cervix (NILM), low-grade precancer (LSIL) to high-grade precancer (HSIL), and eventually invasive cervical cancer stage [19–24].

Studies of vaginal microbiome using the community state type (CST) classification system [25] have observed that four CSTs are dominated by bacteria from the acid producing genus *Lactobacillus*: CST-1 (*L. crispatus*), CST-II (*L. gasseri*), CST-III (*L. iners*), CST-V (*L. jensenii*), while the CST-IV is characterized by diverse groups of anaerobes such as bacteria from the genus *Gardnerella* and other taxa associated with non-optimal cervico-vaginal health [25]. The CSTs have been shown to correlate with severity of cervical precancer and those characterized by depletion of the protective *Lactobacillus crispatus* found in women with high-grades precancer and invasive cervical cancer [26].

Most of these studies have been done in women outside of Nigeria and West Africa. A study examining persistence of low and HR-HPV in HIV positive and HIV negative women in Nigeria found a prevalence of 9% single high-risk HPV in HIV negative women with 4% persistence at 6 months visits, while the baseline prevalence of single HR-HPV was 17% in HIV positive women at baseline with 14% persistence at 6 months [27]. The findings implied that HIV infection was associated with high baseline prevalence of both low- and high-risk HPV, with persistent infection seen in high-risk but not low-risk HPV [27]. One study in Nigeria examined the association of vaginal microbiome and high-risk HPV and found a moderate association between prevalent HR-HPV and a low relative abundance of *Lactobacillus* sp, with a significant abundance of *Prevotella* and *Leptotrichia* in HIV negative women with HR-HPV infection [28]. These findings contribute to the understanding of HR-HPV burden and persistence, and the possible role of vaginal microbiome, but did not relate their findings to the cervical pathology.

We sought to understand the predominant CSTs in Nigerian women and evaluate associations with cervical precancer and invasive cervical cancer, HIV, and HPV infection. We hypothesized that women with high-grade cervical precancer and/or invasive cervical cancer will have more of the non-optimal CSTs and high prevalence of HR-HPV types.

## Methods

### Study setting and design

This was a cross-sectional study of adult women attending the cervical cancer screening and gynecologic oncology unit of the department of obstetrics and gynecology, Jos Teaching Hospital in Jos, North Central Nigeria. Participants were enrolled between January 2020 and February 2022.

### Study participants and data collection procedures

Screening and enrollment of eligible study participants were done at the cervical cancer screening and colposcopy clinic of the Department of Obstetrics and Gynecology of the Jos University Teaching Hospital, Jos, Nigeria. The source population were adult women who presented for cervical cancer screening, follow-up colposcopy for abnormal cytology report, or evaluation for suspected ICC. Women who were at least 30 years old, with no prior treatment for abnormal cervical precancer or invasive cancer and an intact uterus and cervix, and who were not pregnant were eligible for inclusion. Enrollment was stratified by cervical cytology. We enrolled women with a spectrum of negative intraepithelial neoplasia or malignancy (NILM), low-grade squamous intraepithelial lesion (LSIL), high-grade squamous intraepithelial lesion (HSIL), and women with confirmed invasive cervical cancer (ICC). After the principal investigator explained the study purpose and procedures and obtained written informed consent, each participant had a gynecological examination in the colposcopy room. A sterile dry metal speculum was gently inserted into the vagina to visualize the cervix and the vaginal fornices. Hybrid capture 2 Digene HPV collection brushes were used to obtain samples from cervixes, and cytobrushes were used to obtained samples for cytology from each patient. Subsequently, cervico-vaginal lavage samples were collected as described below. The cervical pathological outcomes (NILM, LSIL and HSIL) were categorized and reported according to the Bethesda 2001 system [29], and ICC was diagnosed by histopathology. Details of colposcopic assessments have been described previous reports from Jos Nigeria [3]. Confirmation of HIV status was done in accordance with the HIV Rapid testing algorithm in Nigeria [30]. In brief, HIV testing was done using the national algorithm which included the use of Determine HIV-1/2 as test 1 (T1) (Abbott, California, USA), Unigold HIV-1/2 as test 2 (T2) (Trinity Biotech Plc., Ireland), and StatPak HIV-1/2 as the tie-breaker test (T3) (Chembio Diagnostic Systems, Inc., New York, USA). Individuals that were reactive with T1 and either T2 or T3 were considered HIV-positive [30].

### Laboratory procedures for processing, preservation, and microbiome DNA extraction

#### Sample collection and processing

All sample collections were performed by the gynecological oncology team in the colposcopy assessment room. Cervical vaginal Lavage (CVL) sample was obtained by washing the upper vaginal walls and cervix of each of the female subjects with 10 mL of sterile Phosphate Buffer Saline applied and aspirated with a sterile disposable pipette and Drummond Pipet-Aid. The CVL sample was aseptically aspirated into 15mL sterile falcon tubes placed on ice, capped, and transported to the genomics laboratory for processing and storage within 1–4 h of collection. The 10 mL of CVL sample in the falcon tube was vortexed and aliquoted into 2mL cryovials tubes. The aliquoted 2 mL CVL sample was centrifuged at 10,000 x g for 10 min and 1 mL of the resulting supernatant was pipetted and discarded while the pellet was resuspended in 1 mL of DNA/RNA shield and stored at -80^0^ C.

#### Extraction and purification of DNA

The extraction and purification of the DNA from the CVL sample were performed using Quick-DNA™ Fecal/Soil Microbe Miniprep kit obtained from Zymo Research, USA, following manufacturer directions.

Briefly, the CVL was removed from − 80^0^ C storage and allowed to thaw before it was resuspended by vortexing. 400 µl CVL was used for DNA extraction using the Zymo quick DNA fecal/soil microbe DNA Miniprep kit. Cat No: D6010, Lot No: 207,380, 207,953 &208,889, according to the manufacturer’s protocol. The extracted DNA was quantified using a Qubit dsDNA HS Assay Kit with a Qubit 4 Fluorometer (Thermofisher, Scientific, USA), and stored at -80 °C until use.

#### HPV DNA extraction

As described above the CVL samples were thawed, and 500 µl was pelleted by centrifugation at 3,000 g for 10 min and re-suspended in 200 µl phosphate-buffered saline. DNA was extracted using a QIAamp DNA Mini Kit (Qiagen, Hilden, Germany), following the manufacturer’s protocol. Each DNA extraction run contained positive and negative controls to monitor the extraction procedure and the extracted DNA was eluted in a final volume of 50 µl. The extracted DNA was quantified using a Qubit 4 Fluorometer.

#### Detection of HR-HPV genotypes using the real-time polymerase chain reaction (qPCR)

For each DNA sample, HPV detection and genotyping were performed using Anyplex™ II HPV28 Detection kit (Seegene, Korea). Briefly, each PCR reaction was performed in a 20-µl reaction volume consisting of 5 µl aliquot of DNA added to Anyplex™ PCR Mix (15 µl aliquot each for mixtures A and B) on a CFX96 Real-time PCR system (Bio-Rad Laboratories, Inc., Hercules, CA, USA) according to the manufacturer’s instructions. The thermal cycler conditions consisted of an initial incubation at 50 °C for 4 min, denaturation at 95 °C for 15 min, followed by 50 cycles of denaturation (30 s at 95 °C), annealing (1 min at 60 °C), and elongation (30 s at 72 °C). Cyclic-Catcher Melting Temperature Analysis (CMTA) was performed after PCR cycles 30, 40, and 50. CMTA was performed by cooling the reaction mixture to 55 °C, holding at 55 °C for 30 s, and heating from 55 to 85 °C (5 s/0.5 °C) with continuous fluorescent monitoring. The L1 gene of HPV DNA was the target of the assay, together with simultaneous targeting of a housekeeping gene (Human beta-globin) which was co-amplified as an internal control to monitor DNA purification efficiency, PCR inhibition, and cell adequacy. The results were exported and analyzed using the Seegene Viewer software provided by the manufacturer [31].

### Microbial community characterization

A two-stage PCR protocol was used on the extracted microbiome DNA to amplify the V3-V4 variable region of bacterial 16S rRNA genes, as described previously [32]. Briefly, gDNA was used as template for PCR amplification with the primers CS1_357wF [33] and CS2_806R [34] (ACACTGACGACATGGTTCTACACCTACGGGNGGCWGCAG and TACGGTAGCAGAGACTTGGTCTGGACTACNVGGGTWTCTAAT, respectively; linker sequences underlined). Reactions were performed in 10 µl volumes using repliQa HiFi ToughMix (QuantaBio). Cycling conditions were 2 min denaturation at 98°C, followed by 28 cycles of 98°C for 10 sec, 50°C for 1 sec, and 68°C for 1 sec. Subsequently, a second PCR amplification was performed, also in 10 microliter reactions in 96-well plates using repliQa HiFi ToughMix. Each well received a separate primer pair with a unique 10-base barcode, obtained from the Access Array Barcode Library for Illumina (Fluidigm, South San Francisco, CA; Item# 100–4876). One microliter of PCR product from the first stage amplification was used as template for the 2nd stage, without cleanup. Cycling conditions were 98°C for 2 minutes, followed by 8 cycles of 98°C for 10”, 60 °C for 1” and 68 °C for 1”. Libraries were then pooled and sequenced with a 15% phiX spike-in on an Illumina MiSeq sequencer employing V3 chemistry (2 × 300 base paired-end reads). Library preparation and sequencing were performed at the Genomics and Microbiome Core Facility (GMCF) at Rush University.

Quality control and taxonomic identification and classification into community state types (CSTs) was conducted by University of Maryland Institute for Genomic Science, as described previously [35]. [36]. CSTs were determined by the VALENCIA algorithm implemented by the University of Maryland Institute for Genomic Science, which uses a distance-based metric to classify each sample to a CST based on the similarity of the sample to the centroid of CSTs identified in a reference set. In brief, dominant taxa by CST are as follows: CST-I, *L. crispatus*; CST-II, *L. gasseri*; CST-III, *L. iners*; CST-IV, diverse; CST-V, *L. jensenii.* [36].

### Data management

All data were entered in REDCap and exported as CSV file for coding and subsequent analysis on Stata version 14.1, College station, Texas, USA. The setting and use of REDCap for managing our research data has been reported in an earlier publication [37].

### Stratification of high-risk HPV status

The Seegene AnyplexTM II HPV28 Detection kit detects 28 human papillomavirus types [31].

High-risk HPV (HR-HPV) types include: HPV16, 18, 26, 31, 33, 35, 39, 45, 51, 52, 53, 56, 58, 59, 66, 68, 69, 73, and 82. The low-risk types HPV (LR-HPV) include: HPV6, 11, 40, 42, 43, 44, 54, 61, and 70. The HPV status were categorized as follows: category 1 as HPV negative, category 2 as low-risk risk HPV, category 3 as single high-risk HPV (any of the HR-HPV), and category 4 as multiple high risk (2 or more of the HR-HPV types). For subsequent statistical analysis we recoded HPV types into category 1 as HPV negative; category 2 as low-risk HPV positive, and category 3 as any high-risk HPV positive (single or multiple).

### Statistical analysis

We compared baseline socio-demographic, sexual risk characteristics, CSTs, HPV status, and HIV status between cervical pathology categories using the Pearson’s chi squared or Fisher’s exact tests for categorical variables and ANOVA for normally distributed continuous variables. To assess the association between vaginal microbiome CSTs with grades of cervical pathology, we considered the clinical relevance of the four cervical cytology categories. We combined NILM and LSIL as one group (normal or minor grades dysplasia) while HSIL and ICC were combined as the clinically important group that requires immediate evaluation and treatment. Observations with CST-II (n = 4) were excluded (none was CST-V) from analyses as being too sparse for inference. We performed bivariable logistic regression for the primary exposure variable (CST) and other predictor variables using robust logistic regression to obtain unadjusted odd ratios, 95% confidence intervals (CI), and Wald p-values for associations between HSIL/ICC and NILM/LSIL. We used backward stepwise selection method to build final models checked by the Akaike’s Information Criteria for model selection, including age in years, HIV status, HPV status, CSTs, years of completed education, and total number of births (parity). While tobacco use has been shown to be an important co-factor in cervical cancer risk, only two participants in our sample reported ever smoking, precluding its inclusion in analysis. We then modelled the analyses stratified by high-risk HPV versus negative for HPV (the numbers for low-risk HPV were too small for stratification in the model) to assess the possible effect of CST on cervical pathology probably mediated by high-risk HPV, a known epidemiologic factor in cervical carcinogenesis [2, 38].

## Results

One hundred and fifty-five (155) participants were enrolled, 151 with microbiome data for this analysis (four excluded for having CST-II). The median age of the participants was 52 years (IQR:43–58), 47.7% were HIV positive, and 58.1% had HSIL/ICC. The HR-HPV genotyping results were available in 138 of the participants (the HPV sample of 13 participants were not processed for PCR genotyping and were excluded in all analysis with HR-HPV as an outcome). Of the 138 with HPV data, 56 (40.6%) were negative for HPV, 14 (10.1%) had low-risk HPV, 37 (26.8%) had single HR-HPV, and 31 (22.5%) had multiple HR-HPV types. Of the 31 participants with multiple HR-HPV types, 16 or 18 with any other HR-HPV type were seen in 14 (45.2%), 35 and other HR-HPV types in 4 (12.9%), while other mixed infections other than 16/18/35 were 13 (41.9%). The overall prevalence of any HR-HPV type (single and multiple) was 49.3%, with a higher proportion in women with HSIL/ICC (NILM 31.6%, LSIL 46.5%, HSIL 40.8%, and 81.5% ICC; p = 0.007).

Women with high grade cervical precancer and ICC were on average older than women with NILM and LSIL (Table 1). The distribution of CST showed that 101 participants (66.9%; 95% CI: 58.9–74.0%) had CST-IV. CST-III (*L. iners* dominated) was found in 33 participants (21.9%; 95% CI: 15.9–29.2), while 17 participants had CST-I VMB (11.3%; 7.1–17.4). The distribution of CSTs did not differ significantly by cervical pathology (p = 0.153, Table 1), though cervical cancer diagnosis was more common among women with CST-IV (Table 2). The association of other factors with CSTs as outcome have been summarized in Table 2. HPV status did not significantly differ by CST, though CST-I was more likely among women with HIV infection, and CST-IV was more likely with increased parity.

**Table 1:** Characteristics of participants in comparison to cervical pathology outcomes (N = 151)

Variable	**Cervical Pathology outcome**				P-value
	NILM	LSIL	**HSIL**	**ICC**	
	**N = 19**	N = 45	N = 57	N = 30	
	**n (%)**	**n (%)**	**n (%)**	**n (%)**	
**Vaginal Community State Type (CST)**					0.153
CST-I	2 (11.8)	7 (41.1)	6 (35.3)	2 (11.8)	
CST-III	8 (24.2)	8 (24.2)	14 (42.4)	3 (9.1)	
CST-IV	9 (8.9)	30 (29.7)	37 (36.6)	25 (24.8)	
**HPV status**					0.007
HPV Negative	11 (19.6)	18 (32.1)	24 (42.9)	3 (5.4)	
Low risk HPV Positive	2 (14.3)	5 (35.7)	5 (35.7)	2 (14.3)	
High risk HPV positive	6 (8.8)	20 (29.4)	20 (29.4)	22 (32.4)	
**HIV status**					0.001
HIV Negative	7 (8.6)	36 (44.4)	31(38.3)	7 (8.6)	
HIV Positive	12 (16.2)	10 (13.5)	28 (37.8)	24 (32.4)	
Age (years	48 ± 10.1	48.2 ± 8.5	51.8 ± 9.0	54.3 ± 12.5	0.034
Years of Education completed	12.6 ± 4.2	11.3 ± 4.5	11.3 ± 4.5	7.6 ± 5.7	0.001
BMI (Kg/m^2^)	28.4 ± 5.7	25.6 ± 5.3	25.8 ± 4.3	27.5 ± 7.3	0.163
Age at first sex (years)	18.9 ± 3.6	19.4 ± 4.9	19.7 ± 3.8	17.6 ± 2.3	0.095
Age at first pregnancy(years)	22.2 ± 5.9	20.8 ± 4.3	21.3 ± 4.5	18.5 ± 2.5	0.016
Total life-time number of sex partner(s)	2.8 ± 1.4	3.3 ± 2.4	2.7 ± 2.4	2.5 ± 2.1	0.386
Total years of exposure to hormonal contraception	2.2 ± 4.1	2.9 ± 4.0	2.8 ± 3.9	3.5 ± 6.0	0.858
**Total births (Parity)**					0.007
0–1	5 (29.4)	7 (41.2)	4 (23.5)	1 (5.9)	
2–4	8 (12.1)	22 (33.3)	28 (42.4)	8 (12.1)	
> 4	4 (6.1)	15 (22.7)	26 (39.4)	21 (31.8)	
**Post-menopausal status**					0.262
Yes	10 (18.1)	18 (32.7)	17 (30.9)	10 (18.1)	
No	9 (9.0)	28 (28.0)	42 (42.0)	21 (21.0)	
**Ever drunk Alcohol**					0.322
Yes	1 (3.7)	7 (25.9)	14 (51.9)	5 (18.5)	
No	18 (14.1)	39 (30.5)	45 (35.2)	26 (20.3)	

*Pearson’s chi square test used for comparisons of categorical variables, and Fisher’s exact test used when n < 5 in any cell. For all continuous comparisons ANOVA test was used. Continuous variables are presented as mean plus/minus standard deviation. NILM (Negative for intraepithelial lesion or malignancy), LSIL (low grade squamous intraepithelial lesion), HSIL (high-grade squamous intraepithelial lesion), ICC (invasive cervical cancer). Not all cells sum to N due to missing responses*

**Table 2:** **Distribution of participant characteristics by vaginal microbiome community state types**

Variable	Community state types			P-value
	CST-I	CST-III	CST-IV	
	N = 17	N = 33	N = 101	
**HPV status**				0.114
HPV Negative	12 (21.8)	12 (21.8)	31 (56.4)	
Low risk HPV Positive	0 (0.0)	3 (23.1)	10 (76.9)	
High risk HPV positive	5 (7.6)	14 (21.2)	47 (71.2)	
**HIV status**				0.008
HIV Negative	4 (5.0)	23 (28.8)	53 (66.3)	
HIV Positive	13 (18.3)	10 (14.1)	48 (67.6)	
Age (years)	48.8 ± 11.1	46.9 ± 8.5	52.3 ± 9.9	0.017
Education years completed	13.9 ± 3.5	11.2 ± 3.5	9.9 ± 5.2	0.005
BMI (Kg/m^2^)	30.2 ± 6.9	26.2 ± 5.0	26.0 ± 5.4	0.024
Age first sex (years)	20.9 ± 4.6	18.4 ± 2.6	19.1 ± 4.2	0.105
Age at first pregnancy(years)	22.5 ± 5.1	20.6 ± 4.3	20.5 ± 4.4	0.257
Total life-time sex partner(s)	2.6 ± 2.7	3.4 ± 2.8	2.7 ± 2.0	0.255
Total years of exposure to hormonal contraception	1.9 ± 3.4	2.9 ± 4.1	3.1 ± 4.8	0.681
**Total births (parity)**				0.16
0–1	3 (17.7)	5 (29.4)	9 (52.9)	
2–4	8 (12.3)	18 (27.7)	39 (60.0)	
> 4	4 (6.4)	10 (15.9)	49 (77.8)	
**Current diagnosis of invasive cervical cancer**				0.03
Yes	1 (3.7)	2 (7.4)	24 (88.9)	
No	16 (13.0)	30 (24.4)	77 (62.6)	
**Post-menopausal status**				0.087
Yes	8 (14.8)	16 (29.6)	30 (55.6)	
No	9 (9.3)	17 (17.5)	71 (73.2)	
**Ever drunk Alcohol**				0.897
Yes	2 (7.4)	6 (22.2)	19 (70.4)	
No	15 (12.1)	27 (21.8)	82 (66.1)	

*Pearson’s chi square test used for comparisons of categorical variables, and Fisher’s exact test used when n < 5 in any cell. For all continuous comparisons ANOVA test was used. Continuous variables are presented as mean plus/minus standard deviation. Not all cells sum to N due to missing responses*

**Fig. 1:**
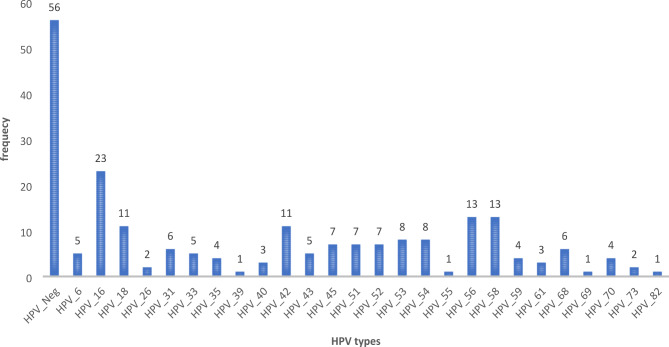
Frequency distribution of type-specific HPV genotypes at enrollment (HPV-16 is the commonest, then types 56,58, 18, followed by 45, 51, 52, and 53)

The distribution of both low and high-risk HPV types in the study sample is shown in Fig. 1 with HR-HPV 16 being the most common. Women with HIV were also significantly more likely to have HSIL/ICC (70.2% vs. 29.3% for women without HIV, p-value = 0.001).

The unadjusted logistic regression (Table 3) showed that age in years (OR = 1.05; 95% CI: 1.01–1.08), positive HIV status (OR = 2.67; 95% CI: 1.38–5.20), and total births (parity) > 4 (OR = 5.94; 95% CI: 1.83–19.3) were significantly associated with HSIL/ICC, while completion of 7–12 years of education (OR = 0.28; 95% CI: 0.09–0.88), or more (OR = 0.29; 95% CI: 0.10–0.85) had significantly lower likelihood for HSIL/ICC. In the final multivariable logistic regression model (Table 3), adjusting for age, HR-HPV status, HIV status, CST, parity, and years of completed education, women with HIV had over three times the odds (aOR = 3.21). Women with any HR-HPV had 2.72 times greater odds of having HSIL/ICC.

**Table 3:** Results of Logistic Regression: Unadjusted and Adjusted odd ratios of the association of various exposure variables with high grades/invasive cervical cancer

Variable	Unadjusted Odds Ratio	95% CI	P-value	Adjusted Odds Ratio	95%CI	P-value
				N = 128		
HPV status						
Low risk HPV Positive	1.1	0.33–3.48	0.905	1.56	0.39–6.26	0.526
High risk HPV positive	1.73	0.84–3.56	0.133	2.72	1.12–6.58	0.027
HPV Negative (referent)						
**HIV status**						
HIV positive	2.67	1.38–5.20	0.004	3.21	1.29-8.00	0.012
HIV Negative (Referent)						
**Vaginal community state type**						
CST-III						
CST-IV	1.2	0.37–3.87	0.766	1.13	0.27–4.67	0.864
CST-I (referent)	1.79	0.63–5.04	0.272	1.31	0.39–4.41	0.666
**Age in years**	1.05	1.01–1.08	0.005	1.01	0.97–1.06	0.483
**Years of Education completed**						
7–12 years						
> 12 years	0.28	0.09–0.88	0.03	0.64	0.14–2.95	0.571
0–6 years (referent)	0.29	0.10–0.85	0.024	0.6	0.14–2.54	0.49
**Parity (total births)**						
2–4	2.88	0.91–9.13	0.072	2.47	0.68-9.00	0.17
> 4	5.94	1.83–19.2	0.003	4.13	0.97–17.7	0.056
0–1 (referent)						

*Covariates included: Age in years, HIV status, HPV status, CSTs, years of completed education, and total number of births*

To further understand the possible associations between CSTs and HSIL/ICC, we stratified our multivariable logistic regression models by HR-HPV status (Table 4). Among women with HR-HPV detected, the effect of HIV was strengthened with aOR = 14.1 (95% CI: 2.34–85.2). It was also observed that the association between CST-III/CST-IV and HSIL/ICC were accentuated in the presence of HR-HPV, although this finding did not reach statistical significance: CST-III (aOR = 6.69; 95% CI: 0.67–66.6) and CST-IV (aOR = 3.64; 95% CI: 0.63–20.9).

**Table 4:** Results of Multivariate logistic regression Stratified by High-risk HPV status

Variable	HPV Negative (N = 53)			High risk HPV positive (N = 62)		
	AOR	95% CI	P-value	AOR	95% CI	P-value
**HIV status**						
HIV positive (vs. HIV negative)	1.6	0.42–6.14	0.493	14.11	2.34–85.2	0.004
Age in Years	1	0.92–1.10	0.839	1.05	0.97–1.15	0.231
**Vaginal community state type (vs. CST-I)**						
CST-III	0.94	0.15-6.00	0.949	6.69	0.67–66.6	0.105
CST-IV	0.89	0.17–4.39	0.864	3.64	0.63–20.9	0.147
**Years of education Completed (vs.0–6 years)**						
7-12years	0.12	0.01–1.94	0.133	1.34	0.22–8.26	0.755
> 12 years	0.27	0.02–3.63	0.322	0.73	0.12–4.49	0.733
**Total births (Parity) (vs 0–1)**						
2–4 births	4.28	0.41–44.74	0.225	2.44	0.41–14.6	0.328
> 4 births	12.05	0.88–164	0.062	1.12	0.13–9.46	0.915

AOR = Adjusted Odds Ratio

## Discussion

This study aimed at understanding the predominant CSTs in Nigerian women and to estimate the associations with cervical precancer and invasive cervical cancer, HIV, and HPV infection found that CST- III and CST-IV vaginal microbiome (VMB) communities were more frequent among women with high-grade cervical precancer and ICC, especially in the presence of HR-HPV. In agreement with a previous report in Nigeria [28], we found that CST-IV was the most frequent in the study sample and did not differ significantly by HR-HPV status, although the former report did not relate the CSTs and HR-HPV to cervical pathology as in our study. Our analyses also confirmed strong associations between HIV/HR-HPV and high-grade cervical precancer or invasive cervical cancer found in several prior studies [2, 39]. Our data showed that women with HIV had 3.2 times greater odds of high-grade cervical precancer and invasive cervical cancer compared to women without HIV. Similarly, we found that the detection of any HR-HPV types increased the odds of high-grade cervical dysplasia and ICC by at least 2.7 times.

In the presence of any HR-HPV types and compared to CST-I (*L. crispatus* dominated), CST-III (*L. iners* dominated) and CST-IV (diverse, non-optimal bacterial species) vaginal microbiomes were associated with high-grade precancer or invasive cervical cancer in a large systematic review and network meta-analysis [40]. Specifically, the meta-analysis showed that vaginal microbiota dominated by non-*Lactobacilli* species (CST-IV) or *L. iners* were associated with 3–5 times higher odds of detecting any prevalent HR-HPV and cervical precancer or invasive cervical cancer compared with a *L. crispatus*-dominated state [40]. Also a study report among South African women assessing incident HR-HPV, vaginal dysbiosis and incident cervical intraepithelial neoplasia grade 2 or more (CIN 2+) found that the acquisition of HR-HPV changes the vaginal microbiome and that anaerobic dysbiosis risk seem to increase concurrently with development of CIN2 + suggesting that the role of the vaginal microbiome composition in CIN2 + development could be mediated by persistent HR-HPV infection [41]. Our findings are therefore in agreement with the literature implying that a better understanding of the cervico-vaginal microenvironment could be a key step for HPV viral clearance [42]. This mechanistic role of the cervico-vaginal microbiome community state types is critical and could be explored in developing therapeutic targets for cervices already infected with the HR-HPV types but yet to advance the process of cervical carcinogenesis given the high prevalence of these common sexually transmissible viral infection in the general population. Currently, the primary HPV preventive vaccines are not readily available or affordable in most LMICs making the search for therapeutic options for cervical HPV infection a promising effort.

Furthermore, the associations between CST and the two sexually transmissible viruses - HR-HPV and HIV - have public health implications for cervical cancer prevention. In 2018, the World Health Organization launched a global initiative to eliminate cervical cancer as a public health problem by adopting highly effective evidence-based interventions 2030 [14]. These interventions includes increasing the population-level coverage for HPV vaccination of 90% of eligible young girls between 9 and 15 years; screening of 70% eligible women by 35 years and 45 years, and effective treatment of 90% women with precancer and invasive cervical cancer by 2030 [14]. As discussed earlier, most countries in LMICs including Nigeria do not have a national program on HPV vaccination [43], making interventions for therapeutics, and HIV prevention, and to maintain a healthier cervico-vaginal environment effective and plausible public health approaches for cervical cancer prevention and control efforts. Indeed, some of the known epidemiologic factors for cervical cancer [15, 24, 44–54] such as early sexual debut, multiple sexual partners, smoking, and unsafe sexual practices are modifiable and could be controlled using public health approaches. To further support this, the Global Burden of Cancer attributable to modifiable risk factors confirmed the contribution of behavioral risk factors such as smoking, alcohol use, and unsafe sex as modifiable risk factors attributable to a huge burden of cancer in the population [54]. Achieving population level changes in unsafe sexual practices, however, will require societal efforts in addressing the structural and system drivers of early sexual debut and multiple sex partners such as economic, legal, cultural, and religious beliefs. In the area of therapeutics, it is possible that the biological interplay of CSTs, HR-HPV, and HIV immunosuppression seen in our study could be further examined to develop point-of-care molecular testing for rapid detection and quantification of *Lactobacillus* and other non-*Lactobacillus* dominant cervico-vaginal microbiota as biomarkers for evaluation, early detection, and treatment of high-grade precancer in the population.

Our study also showed the importance of socioeconomic factors, such as more years of completed education was associated with lesser odds for high-grade cervical precancer/ICC, while age and higher number of total births (parity) were associated with greater odds for high-grade cervical precancer/ICC. Furthermore, the unadjusted analyses of age at first pregnancy showed a significant relationship favoring women whose first pregnancies were at relatively younger age (See Table 1), and these women were more likely to have a CST-IV (Table) compared to women who were older at first pregnancy. The epidemiologic findings seem to be in agreement with recent genome-wide association study which showed that women who were older at first pregnancy had a lower odds for ICC [55]. The interplay of these sociodemographic variables in association with unfavorable CSTs and in predicting high grades precancer and/or invasive cervical cancer is an interesting finding particularly in sub-Saharan Africa where educational attainment is low, and lower educational status has been shown to be associated with higher number of births [56]. As shown in Table 4, there is a greater odds for high-grade cervical precancer or ICC if parity is > 4 in women with negative HR-HPV. The epidemiologic nuances surrounding this relationship, and the mechanism by which repeated childbirths contributes to cervical carcinogenesis requires further investigation particularly in the absence of HR-HPV given emerging ideas that certain sub-types of cervical cancer are not caused by HPV [57, 58].

Our data revealed trends that need confirmatory longitudinal studies with larger sample size to further characterize biomarkers of the vaginal microbiome and HR-HPV detection in Nigerian women at various stages of cervical precancer to understand how they predict progression to high-grade precancer or invasive cervical cancer stages. We intuitively hypothesized that therapeutic approaches that modifies and maintains a more optimal cervico-vaginal microenvironment could reduce the acquisition or help the clearance of HR-HPV in cervices before the process of HPV-induced cervical carcinogenesis advances to high-grades precancer and invasive stages. This would be a highly translatable public health research effort and holds the prospect for reducing population level incidence of cervical precancer and/or invasive cervical cancer particularly in Nigeria and other LMICs where primary preventive HPV vaccines and cervical cancer screening coverage are still very low.

Our study findings have a few limitations, mainly in the relatively small sample size and cross-sectional design. We also lacked the data on duration for HPV infection and did not have data on HIV treatment with antiretrovirals, viral load, or immune suppression levels with CD4 count markers. We also did not have control on intravaginal practices such as douching with soap and water, and other agents on vaginal microbiome as previously reported in the literature [59]. Nonetheless, our findings, the first of their kind from Nigeria characterizing vaginal microbiome and relating it to HR-HPV and spectrum of normal cervix, low and high-grades precancer and invasive cervical cancer pathology, suggest a potential relationship between CST, HR-HPV, and progression to high-grade precancer and/or ICC. The findings also support the well-known evidence that HR-HPV is the principal causative factor [1, 2, 60] in cervical precancer and progression to invasive cervical cancer and add to the literature in the paucity of this information from Nigeria and sub-Saharan Africa.

## Conclusions

We conclude that non-optimal CSTs, particularly when HR-HPV is detected, may assist in identifying women with high-grade cervical precancer. Characterization of CST using point-of-care molecular testing particularly in women identified with any HR-HPV may be a useful biomarker for early detection of severe precancer and cervical cancer prevention. Longitudinal data will improve our understanding of the temporal effect of non-optimal CSTs, HR-HPV, and other factors in cervical cancer development, and may lead to translatable public health interventions towards cervical cancer prevention and elimination globally.

## Data Availability

All the relevant data for this analysis have been presented in the body of this manuscript and the associated tables and figures. The original database of the phenotypic data is available in the institutional REDCap database of the University of Jos. The microbiome genomic raw data has been submitted to the Sequence Read Archive (SRA) database at the NCBI with Accession Number: PRJNA914231.
